# Association Between Habitual Dietary Intake and Urinary Metabolites in Adults—Results of a Population-Based Study

**DOI:** 10.3390/metabo15070441

**Published:** 2025-07-01

**Authors:** Annika Blümlhuber, Dennis Freuer, Nina Wawro, Florian Rohm, Christine Meisinger, Jakob Linseisen

**Affiliations:** 1 Epidemiology, Faculty of Medicine, University of Augsburg, 86156 Augsburg, Germany; 2 Institute for Medical Information Processing, Biometry and Epidemiology—IBE, LMU Munich, 81377 Munich, Germany; 3 Pettenkofer School of Public Health, 81377 Munich, Germany; 4 Institute of Epidemiology, Helmholtz Center Munich, 85764 Neuherberg, Germany; 5 Institute of Epidemiology, University Hospital Augsburg, Stenglinstr. 2, 86156 Augsburg, Germany

**Keywords:** urinary metabolites, habitual dietary intake, metabolomics, dietary biomarkers

## Abstract

Background: Chronic non-communicable diseases (NCDs) are a major global health challenge, with unhealthy diets contributing significantly to their burden. Metabolomics data offer new possibilities for identifying nutritional biomarkers, as demonstrated in short-term intervention studies. This study investigated associations between habitual dietary intake and urinary metabolites, a not well-studied area. Methods: Data were available from 496 participants of the population-based MEIA study. Linear and median regression models examined associations between habitual dietary intake and metabolites, adjusted for possible confounders. K-means clustering identified urinary metabolite clusters, and multinomial regression models were applied to analyze associations between food intake and metabolite clusters. Results: Using linear regression models, previously reported associations could be replicated, including citrus intake with proline betaine, protein intake with urea, and fiber intake with hippurate. Novel findings include positive associations of poultry intake with taurine, indoxyl sulfate, 1-methylnicotinamide, and trimethylamine-N-oxide. Milk substitutes were positively associated with urinary uracil, pseudouridine, 4-hydroxyhippurate, and 3-hydroxyhippurate, and inversely associated with quinic acid. Dietary fiber intake showed a positive association with 3-(3-hydroxyphenyl)-3-hydroxypropionic acid and a negative association with indoxyl sulfate. We identified sucrose and taurine as key metabolites differentiating metabolite clusters. Multinomial regression analysis confirmed significantly different dietary associations across clusters, particularly for fruits, processed meat, poultry, and alcoholic beverages. Conclusions: This study highlights established and novel food–metabolite associations, demonstrating the potential of urinary metabolomics for use as nutritional biomarkers in individuals from the general population.

## 1. Introduction

Chronic non-communicable diseases (NCDs) are the leading global causes of morbidity, disability, and healthcare expenditures, accounting for nearly two-thirds of all deaths annually [1,2,3]. Each year, NCDs cause 41 million deaths, primarily due to cardiovascular diseases, cancers, chronic respiratory diseases, and diabetes [4]. Between 2000 and 2019, global NCD-related deaths rose from 59.5% to 73.9%, underscoring their growing impact [5]. Unhealthy diets, alongside physical inactivity, smoking, and alcohol consumption pattern, are key drivers of NCDs, influencing metabolic pathways like inflammation, oxidative stress, and derangement in lipid metabolism [6]. Understanding the impact of dietary factors on health is therefore essential to improve dietary practices and reduce the global burden of NCDs.

As dietary assessment is time-consuming and prone to bias, biomarkers have emerged as an essential tool for supplementing or even replacing dietary assessment; if successfully established, they provide objective and reliable measures of intake and nutrient status and are not suffering from biases of self-reported data, such as recall errors and social desirability [7,8,9]. Building on this, metabolomics captures a wide range of metabolites influenced by genetic, environmental, and dietary factors, providing deeper insights into the complex interplay between diet and metabolic health [10,11].

Despite advancements in nutritional research, most metabolomics studies focused on controlled (short-term) dietary interventions, limiting their relevance for real-world dietary data. Habitual intake, i.e., long-term dietary intake, remains underexplored due to challenges in capturing data under real-world conditions [12,13]. Urine-based metabolomics addresses this gap by offering a comprehensive metabolic overview, encompassing metabolites from food, the microbiome, and endogenous processes. Its ability to detect food-derived metabolites, often less abundant in plasma, makes it particularly effective for studying habitual dietary intake [12]. However, validating urinary biomarkers for specific foods and dietary components remains challenging, as most have been identified under controlled conditions with limited relevance to diverse, free-living populations [14]. Our study aimed to overcome these limitations by analyzing habitual dietary intake and urinary metabolites in the population-based “Metabolism, Nutrition and Immune System in Augsburg” (MEIA) study.

## 2. Materials and Methods

### 2.1. Study Design and Population

The present study used data from the MEIA study, a population-based study conducted in the Augsburg region to explore associations between nutrition, metabolism, and immune function. A random sample from civil registries stratified by age and sex was selected and invited to participate. Potential participants were contacted via postal invitations and followed up with telephone reminders. Eligible participants were adults aged 19–75 years, residing in the Augsburg region, i.e., in the city of Augsburg and the surrounding counties of Augsburg and Aichach-Friedberg. Exclusion criteria included serious health conditions or inability to provide informed consent. Recruitment and data collection occurred between April 2021 and July 2023. Among the 594 participants enrolled in the MEIA study, 496 had complete data on dietary intake—defined as having responded to all dietary intake questions without any missing values—and were included in the present analysis. The MEIA study was performed in accordance with the Declaration of Helsinki. Ethical approval was obtained from the Ethics Committee of the Ludwig-Maximilians-Universität München. All participants provided informed written consent before participation.

### 2.2. Data Collection

Participants from the MEIA study were examined at the study center of the Chair of Epidemiology at the University Hospital Augsburg. During their visit, participants completed a computer-assisted personal interview. The interview collected detailed information on medication use, health status, and lifestyle factors. Additionally, participants completed a computer-assisted questionnaire on a tablet, which covered dietary supplement use, eating behavior, smoking habits, healthcare utilization, and preventive health measures. Dietary intake was assessed using the myfood24 online platform as described in detail below [15]. In addition to dietary assessments, participants underwent physical, cognitive, and anthropometric measurements during their study visits. Biological samples, including fasting venous blood, spot urine, and fecal samples, were collected. Blood samples were analyzed for routine laboratory parameters such as blood lipids and liver enzymes. All samples were processed and stored at −80 °C in the biorepository of the Chair of Epidemiology for future analyses. In the present analysis, we focused on spot urine samples for metabolomics analysis.

### 2.3. Dietary Assessment

To evaluate dietary intake, the myfood24 online tool (Dietary Assessment Ltd., Leeds, UK) was utilized, specifically designed for large-scale epidemiological studies. Myfood24 was chosen for its ability to efficiently capture detailed dietary data, offering a time-saving and less administratively demanding alternative to traditional methods. At the study center, participants were introduced to myfood24 through a brief video tutorial before recording their dietary intake from the previous day. Following the initial dietary assessment, participants were asked to complete three additional 24-h dietary recalls over the next six weeks. These recalls were scheduled on randomly selected weekdays and weekends to capture variations in daily intake patterns. Participants received e-mail invitations with links to myfood24 on the specified days. As an alternative to self-reported online diet assessment, 11.6% of participants preferred to complete 24-h diet recalls by phone (assisted by a trained interviewer). A portion size guide, containing all available options in myfood24, was provided to aid in accurate reporting during telephone interviews. To capture additional information about eating contexts, participants were asked questions on the location and social context of each meal. The usual dietary intake was derived as a weighted mean over the observed 24-h recalls available for the participant. The initial recall conducted at the study center was excluded due to the 12-h fasting requirement. Only participants with at least two additional recalls (i.e., a total of three or four) were included. Ideally, information from two weekdays and one weekend day (i.e., from Saturday or Sunday) should be collected. To account for potential differences in eating behavior between weekdays and weekends, we applied a weighted means approach, assigning a weight of 5/7 to weekdays and 2/7 to the weekend day. This approach was based on previous literature indicating systematic differences in dietary intake across the week [16]. While the use of multiple 24-h recalls per individual is a common approach to characterize habitual intake, with-in person variability often leads to distorted intake distributions. To address this challenge, several statistical methods have been developed to better describe usual intake distributions [17,18]. Since our focus was on analyzing individual-level associations rather than estimating population-wide intake distributions, the weighted means approach was chosen as an appropriate and pragmatic method for approximating individual habitual intake [16,19,20]. Participants with an energy intake–to–basal metabolic rate ratio below 0.6 were excluded as extreme underreporters. Basal metabolic rate was estimated using the Schofield equation based on sex, age, and body weight [21]. The food items were grouped into meaningful food groups and subgroups.

### 2.4. Assessment of Urinary Metabolites

Spot urine samples (20 mL) were collected from participants during their visit to the study center after an overnight fast of at least 12 h and used for metabolomics analysis. The urine samples were processed at the laboratory of the Chair of Epidemiology at the University Hospital Augsburg. After centrifugation and aliquoting, the samples were stored at −80 °C until analysis. Laboratory analysis was performed at Nightingale Health, Helsinki, Finland, using a high-throughput nuclear magnetic resonance (NMR) spectroscopy platform optimized for quantifying abundant urinary metabolites with minimal signal overlap [22]. Urinary metabolite concentrations were expressed relative to creatinine, reported in mmol/L, and scaled by a factor of 100 to enhance interpretability. Missing values were imputed using half the lowest detected metabolite concentration, representing the detection limit.

### 2.5. Covariables

Covariables were selected based on the established literature [12,23] and included: age (in years), sex, body mass index (BMI) (in kg/m^2^), physical activity level (PAL), smoking status, and alcohol consumption pattern. Body weight and height were measured in light clothing and used to calculate the BMI. PAL was assessed using the European Health Interview Survey-Physical Activity Questionnaire (EHIS-PAQ), classifying activity according to participant-reported frequency, duration, and intensity [24]. These responses were then used to categorize PAL (sedentary, low active, active, very active) [25]. Smoking status was classified from questionnaire responses as current smoker, former smoker, or never smoker, based on self-reported smoking history. Alcohol consumption pattern was assessed using the AUDIT-C score, a validated screening tool that evaluates patterns of alcohol use on a scale from 0 to 12. Higher scores reflect a more frequent or risk-associated alcohol consumption pattern, but not necessarily a higher absolute intake. Risk levels were categorized as low for scores between 0 and 2 in women and 0 and 3 in men, moderate for scores of 3 to 5 in women and 4 to 5 in men, high for scores of 6 to 7 in both sexes, and severe for scores ranging from 8 to 12 in both sexes [26].

### 2.6. Statistical Analysis

The analysis focused on 49 out of 51 urinary metabolites; urea was excluded from cluster analyses due to its broad range and disproportionately high values, which could distort clustering results, and creatinine was excluded because all metabolites were already normalized to urinary creatinine. Both metabolites, however, were retained for selected individual analyses. Patient characteristics, food intake, and urinary metabolite data were summarized using both means and standard deviations (SDs) as well as medians and interquartile ranges (IQRs) for continuous variables. Categorical variables were described using absolute and relative frequencies. We applied Student’s t-tests for normally distributed continuous variables, Mann–Whitney U-tests for non-normally distributed continuous variables, and Chi-square tests for categorical variables. Corresponding *p*-values were used to evaluate statistical significance. Most metabolite concentrations were log2-transformed to address skewness and outliers. Exceptions were made for 2-hydroxyisobutyrate, ethanolamine, pseudouridine, and urea, which followed approximately normal distributions and were not log-transformed. Normality assumptions were evaluated visually using Q-Q plots and statistically assessed with the Kolmogorov–Smirnov test. To test the linearity assumption, restricted cubic splines for continuous predictors were applied, with the optimal number of knots (3–5) determined using the Akaike Information Criterion (AIC). Homoscedasticity and the normal distribution of residuals in parametric models were visually assessed using residual scatter plots and Q-Q plots, respectively.

Linear regression models were applied to explore associations between individual food groups, subgroups, or selected nutrients (exposure) and specific urinary metabolites (outcome). Median regression was performed as an alternative statistical method to provide more robust estimates when the assumptions for linear regression could not be met. For these models, standard errors were estimated using bootstrap. In contrast, standard errors in linear regression models were derived using conventional model-based estimation. Both linear and median regression models were adjusted for potential confounders, including age, sex, BMI, PAL, smoking status, and alcohol consumption pattern. Interaction terms were tested to evaluate effect modification by sex and age on the relationship between predictors and metabolite outcomes. 

Clustering analysis was performed to identify patterns of urinary metabolites. A series of cluster analyses were performed using different cluster sizes (K = 2–8). 30 cluster validity criteria from the R package “NbClust” (version 3.0.1) were applied to determine the most appropriate number of clusters. The optimal solution was determined to be four clusters. K-means clustering was applied to group data into clusters, iteratively updating centroids to minimize within-cluster variance. This process continued until the clusters were stable. Cluster 1 (*n* = 10) was excluded from further analysis due to its small sample size and low metabolite concentrations. This pattern is likely attributable to a high proportion of imputed values, as missing metabolite concentrations were replaced using the respective detection limits. These preprocessing-related effects may have artificially driven the formation of this separate cluster. The analysis continued with three clusters, where Cluster 2 included 84 participants, Cluster 3 included 56 participants, and Cluster 4 included 346 participants. This resulted in a total sample size of 486 participants, which was consistently used in all subsequent analyses, including linear and median regression models. The identified clusters served as the basis for subsequent analyses exploring their association with dietary intake.

Further, multinomial regression models were employed to assess associations between intakes of individual food groups (exposures) and clusters of metabolites (outcomes). Variable Importance in Projection (VIP) values were calculated to assess the contribution of each food group, with values above 1 considered significant. The VIP threshold was set at 1, rather than the standard 1.5, to include a broader range of food groups in the analysis. A higher threshold would have excluded several relevant groups, limiting the comparative analysis. Subcategories of food groups that were identified as significant through multinomial regression were also incorporated into the analysis. In total, 16 food groups and subgroups were considered in the analysis, including meat, vegetables, milk and dairy products, fish and seafood, plant-based substitutes, and alcoholic beverages (including beer, wine/sparkling wine, spirits, and liqueurs/cocktails). Additional categories included milk, fermented dairy products, cheeses other than fresh cheese, milk substitutes, as well as dietary fiber and whole grain products. Multicollinearity among the selected predictors (food groups) was evaluated using the Variance Inflation Factor (VIF), and all values remained below 5.0, indicating no notable multicollinearity issues. Statistical significance was defined as *p*-values < 0.05. All statistical analyses were performed using R software (version 4.4.1).

## 3. Results

### 3.1. Study Population

Participant characteristics for the total sample and stratified by sex are presented in Table 1. Of the 496 participants included in the urinary metabolomics analysis, 211 (42.54%) were male and 285 (57.46%) were female. The median age of the total sample was 49.00 ± 14.64 years, with no significant difference in age distribution between males and females (*p* = 0.430). However, significant differences were identified in several metabolic and dietary variables between the sexes. Men had significantly higher BMI and waist circumference compared to women. HDL cholesterol levels were significantly higher in women compared to men. Behavioral factors, such as smoking habits and PAL were similar between men and women. Men were more likely to be classified as high- or severe-risk alcohol misusers compared to women (*p* < 0.001). Habitual dietary intake data based on repeated 24-h dietary recalls is presented in Table 2. Men reported significantly higher consumption of processed meat, edible fats/oils other than butter, bread and bakery products, alcoholic beverages, and meat and milk alternatives. Women consumed significantly more vegetables, non-alcoholic beverages, and fruits. Table 3 shows urinary metabolite concentrations stratified by sex. Significant differences were observed for several metabolites. For example, men exhibited higher concentrations of allantoin, creatinine, 4-deoxythreonate, taurine, and tyrosine, while women showed higher levels of acetate, arabinose, dimethylamine, ethanolamine, ethanol, formate, glucose, glycine, hippurate, and 3-(3-hydroxyphenyl)-3-hydroxypropionic acid. 

### 3.2. Individual Associations Between Habitual Food Intake and Urinary Metabolites

Table 4 presents the association of habitual food or nutrient (dietary fiber) consumption with urinary metabolites, adjusting for age, sex, BMI, PAL, smoking status, and alcohol consumption pattern. Consistent with prior findings, significant positive associations were observed between citrus intake and proline betaine (ß = 0.005, 95% CI 0.001–0.009, *p* = 0.006), protein intake and urinary urea levels (ß = 0.078, 95% CI 0.028–0.129, *p* = 0.002), and dietary fiber intake and hippurate (ß = 0.013, 95% CI 0.006–0.021, *p* < 0.001). In addition, several new associations were identified. Poultry consumption was found to be positively associated with multiple metabolites, including taurine, indoxyl sulfate, 1-methylnicotinamide, and trimethylamine-N-oxide. Significant associations were also observed between milk substitute consumption and urinary levels of uracil, pseudouridine, 4-hydroxyhippurate and 3-hydroxyhippurate; additionally, a significant inverse association was found for quinic acid. Dietary fiber intake was positively associated with 3-(3-hydroxyphenyl)-3-hydroxypropionic acid and hippurate. A negative association was observed between indoxyl sulphate and dietary fiber intake. No clear interaction effects with age and sex were identified in the linear and median regression models.

### 3.3. Description of Identified Metabolite Clusters 

**Figure 1:**
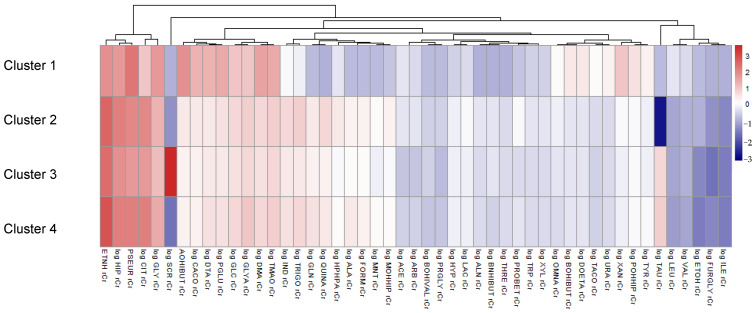
Heatmap of standardized median metabolite concentrations across clusters. Clusters were identified using k-means.

We stratified the study population into metabolite clusters to explore potential differences in their associations with dietary behavior. No significant difference in age distribution was observed across clusters. However, participants significantly differed by sex (*p* < 0.001), with Cluster 3 having the highest proportion of males (67.86%), followed by Cluster 4 (44.22%). Conversely, the highest prevalence of females was observed in Cluster 1 (80.00%), followed by Cluster 2 (78.57%) and Cluster 4 (55.78%) (Supplementary Table S1). In addition to demographic differences, clusters exhibited significant differences in metabolic and dietary parameters, most notably in HDL cholesterol levels (*p* = 0.027), protein intake (*p* = 0.019), fat consumption (*p* = 0.032), and total energy intake (*p* = 0.033). Cluster 3 consistently showed higher energy intake, while Clusters 1 and 2 exhibited a significantly lower mean energy intake. In addition, processed meat consumption was significantly higher in Cluster 3 compared to Cluster 1, Cluster 4, and Cluster 2 (*p* = 0.002). Concerning metabolites, the strongest differences between clusters were identified for urinary sucrose and taurine levels, as illustrated in the heatmap in Figure 1. Cluster 1, which contained only 10 participants, was characterized by, e.g., higher levels of 2-hydroxyisobutyrate, cis-aconitate, 4-deoxythreonate, pyroglutamate, or glucose and lower levels of indoxyl sulfate, trigonelline, glutamine, and quinic acid, when compared to Clusters 2, 3, and 4. Significant differences across the remaining three clusters were observed for several metabolites, including alanine, acetate, arabinose, 3-hydroxyisovalerate, cis-aconitate, creatine, dimethylamine, 4-deoxythreonate, 2-furoylglycine, isoleucine, 3-hydroxyhippurate, pyroglutamate, pseudouridine, sucrose, taurine, trigonelline, tyrosine, and valine (*p* < 0.05) (Supplementary Table S2). Overall, the concentrations of urinary metabolites were found to be largely similar across Clusters 2, 3, and 4.

### 3.4. Association of Habitual Food Intake and Clusters of Urinary Metabolites

After excluding Cluster 1, the associations between 16 food groups and subgroups with Clusters 2, 3, and 4 were analyzed using multinomial logistic regression models. Two multinomial models were used, with either cluster 2 or cluster 4 as the reference category. Overall, dietary intake patterns were broadly comparable across clusters, with limited variation in the consumption of most food groups. However, significant linear associations were observed for alcoholic beverages between Cluster 3 and Cluster 4 and between Cluster 3 and Cluster 2, with the strongest association observed for beer consumption (Supplementary Table S3). In contrast, non-linear associations were found for fruit intake between Cluster 3 and Cluster 4, for poultry intake between Cluster 3 and Cluster 2 as well as between Cluster 2 and Cluster 4, as well as for processed meat intake between Cluster 3 and Cluster 4 (Supplementary Figures S1–S4).

## 4. Discussion

In this study, we explored the associations between habitual dietary intake and urinary metabolites in 486 adults from a population-based sample. Our findings for specific food–metabolite associations replicated several known associations while also revealing novel diet–metabolite relationships. Additionally, we identified four clusters of metabolites using K means, but differential relationships with habitual food consumption were limited.

Our results confirm three previously established food–metabolite associations, including citrus fruit consumption, protein intake, and dietary fiber intake. Proline betaine, a well-established biomarker of citrus intake, has been validated in both controlled feeding and population-based studies. It is considered a reliable short-term and habitual marker, with evidence of high sensitivity (90.6%) and specificity (86.3%) in intervention studies, as well as increased urinary excretion among habitual citrus consumers [27,28]. In our analysis, we also found a significant positive association between citrus intake and urinary proline betaine levels, providing further support for its reliability as a biomarker of habitual citrus fruit consumption. Although the direct health effects of proline betaine remain unclear, its association with diets high in fiber and low in sodium and fat suggests potential cardiovascular benefits [29,30]. Another significant association described in previous studies was observed between protein intake and urinary urea levels. Urea, the primary nitrogen carrier in urine, has been shown to increase with higher protein intake [31,32,33,34]. It is produced in the liver from nitrogen released during amino acid metabolism and is excreted in the urine, making its urinary concentration a common and well-established marker in metabolomics studies to assess dietary protein intake [32]. This relationship has been documented in both experimental and population-based studies. For instance, the INTERMAP Study included detailed dietary assessments and urinary biomarker analysis across diverse international cohorts, confirming urinary urea as a reflection of protein intake [33]. In addition, physiological reviews and experimental studies on nitrogen metabolism support this relationship from a mechanistic perspective [31,32,33]. Fiber intake was associated with urinary hippurate, a marker derived from polyphenol-rich foods such as fruits, vegetables, tea, and wine [35,36,37,38,39,40]. The validity of hippurate as a biomarker for polyphenol and fiber intake has been demonstrated in both controlled feeding and population-based studies. Higher hippurate levels indicate increased polyphenol and dietary fiber intake, which has been confirmed in different populations [36,41]. Beyond its role as a dietary marker, hippurate is associated with increased gut microbiome diversity, reduced risk of metabolic syndrome, and improved glucose tolerance [42,43]. The specificity of taurine and trimethylamine-N-oxide (TMAO) as a red meat biomarker remains inconclusive, as we found no significant association, consistent with other studies suggesting 1- and 3-methylhistidine as more reliable markers [44,45,46,47,48]. Additionally, unlike prior research [49,50,51], we found no association between fish intake and urinary TMAO, dimethylamine, or taurine.

Our analysis identified novel associations between poultry consumption and several urinary metabolites, including taurine, indoxyl sulphate (IS), 1-methylnicotinamide (N1-MN), and TMAO. Taurine, abundant in animal tissues and particularly rich in poultry, eggs, and dairy products, was identified as a marker of poultry intake in our study, differing from broader associations with omnivorous diets reported in previous studies [52,53]. This specificity may be influenced by endogenous taurine synthesis, as even low intake groups, such as vegans, can still excrete measurable taurine concentrations, potentially explaining inconsistencies of taurine as a marker of meat consumption [54]. Poultry consumption was also significantly associated with urinary IS, a toxin formed by the hepatic metabolism of indole, which is produced via gut bacterial fermentation of dietary tryptophan [55]. Given poultry’s high tryptophan content, higher consumption may elevate urinary IS levels, though further investigation is needed [56,57]. In addition, N1-MN, a niacin metabolite influenced by niacin and tryptophan intake, was positively associated with poultry, consistent with prior findings, linking niacin-rich foods such as poultry to elevated N1-MN levels [58,59,60]. Our findings further identified TMAO, a metabolite formed from compounds such as choline and carnitine found in animal foods and metabolized by gut bacteria, to be associated with poultry intake [49,61]. However, research on TMAO has shown mixed results, with Dai et al. [49] reporting a negative association, suggesting poultry may contribute less to TMAO formation than other animal proteins. These discrepancies likely reflect interindividual differences in gut microbiota composition and metabolic activity, which are known to influence the microbial conversion of precursors into trimethylamine, the immediate precursor of TMAO. In addition, hepatic expression of flavin-containing monooxygenase 3, which oxidizes trimethylamine into TMAO, can vary due to genetic polymorphisms and host metabolic status. Finally, overall dietary patterns, including fiber intake and the presence of other TMAO-rich foods such as fish, may further modulate TMAO production and clearance [50,61,62,63]. These factors highlight the complex interplay between diet, host metabolism, and microbial composition in determining TMAO levels. Furthermore, we identified associations between dairy alternatives and several urinary metabolites, including uracil, pseudouridine, 4-hydroxyhippurate, 3-hydroxyhippurate, and quinic acid. Milk substitute consumption was positively associated with urinary levels of uracil, a pyrimidine base associated with soy products, particularly fermented soy milk. Uracil’s bioactive effects, such as angiotensin-converting enzyme (ACE) inhibition and potential skin benefits, suggest its role in the health benefits of fermented soy products [64,65]. Pseudouridine, a modified nucleoside associated with ribonucleic acid (RNA) turnover and often elevated in oncogenic conditions, has also been positively associated with milk substitute consumption [66,67,68]. Given its role in cellular and metabolic regulation, its relation to milk substitute intake may reflect increased RNA metabolism, although its association with milk substitutes does not necessarily indicate a unique dietary source. Our analysis additionally indicated that 4-hydroxyhippurate, typically derived from polyphenol-rich foods, was positively associated with milk substitutes. This suggests that soy may play a role in polyphenol metabolism and excretion. This finding is consistent with the results of previous studies, e.g., by Jacobs et al., which demonstrated an increase in urinary 4-hydroxyhippurate in participants who consumed polyphenols with soy milk. This highlights the efficacy of soy as a carrier of polyphenols [69]. Additionally, 3-hydroxyhippurate has been shown to exhibit a notable positive correlation with milk substitutes. Similar to 4-hydroxyhippurate, it has been previously identified at elevated levels in urine after polyphenol intake. Nevertheless, a direct relationship between this substance and milk substitutes has yet to be documented in the literature [70]. Furthermore, a significant negative correlation was identified between milk substitute consumption and urinary quinic acid levels. Quinic acid, abundant in coffee, fruits, and vegetables, is metabolized by gut bacteria into 4-hydroxyhippuric acid, a biomarker for polyphenol intake [71,72]. The inverse association may reflect the differing effects of milk substitutes on gut microbiome functionality compared to traditional dairy products, which in turn affect polyphenol and quinic acid metabolism [73,74]. In addition to poultry and milk substitute consumption, our analysis revealed novel associations between dietary fiber intake and various urinary metabolites, including a positive association with 3-(3-hydroxyphenyl)-3-hydroxypropionic acid (HPHPA) and an inverse association with IS. No significant associations were observed with several amino acid-derived compounds. HPHPA, an organic acid commonly found in urine, is derived from polyphenols and phenylalanine and can also be produced by gut microbial metabolism [75,76,77]. Although a direct link between fiber intake and urinary HPHPA has not been widely documented, a related metabolite, 3-(2-hydroxyphenyl)-propionic acid (2-HPPA), has shown positive associations with fiber intake in recent studies [37]. IS, linked to tryptophan metabolism, showed an inverse relationship with fiber intake, consistent with studies suggesting fiber’s role in reducing IS levels by improving gut health [55,78]. Acetate, a short-chain fatty acid (SCFA) associated with gut microbial fermentation of fiber, showed limited urinary association likely due to its rapid absorption [79,80,81,82]. Despite the influence of fiber on gut microbiota and amino acid metabolism, metabolites such as 3-aminoisobutyrate, 3-hydroxyisobutyrate, 2-hydroxyisobutyrate, 3-hydroxyisovalerate, and formate showed no significant associations, as they are primarily associated with amino acid and one-carbon metabolism rather than fiber intake [79,83,84,85,86].

Urine’s ease of collection, minimal interference from other compounds, and rapid response to dietary changes make it particularly suitable for identifying dietary biomarkers and studying metabolic health [87,88]. Its high concentrations of organic acids and microbial metabolites provide valuable insights into dietary behavior and metabolic health [89]. Gut microbial metabolites in urine are significantly associated with chronic diseases, with the gut microbiota converting dietary fiber into bioactive compounds like SCFAs (acetate, propionate, and butyrate), which improve gut health and reduce the risks of obesity, metabolic syndrome, and type 2 diabetes [90]. These metabolites are classified into three types: (1) diet-derived compounds like SCFAs and indole derivatives; (2) host metabolites modified by microbiota, such as secondary bile acids; and (3) de novo microbial products, like polysaccharide A [89]. By capturing complex diet-gut interactions, urinary metabolites serve as valuable biomarkers to assess the impact of diet on chronic and communicable diseases. 

Building on these insights into diet–metabolite associations, our study applied cluster analysis to uncover patterns in urinary metabolites, using k-means to build clusters independent of any endpoint. Unlike conventional targeted approaches such as partial correlation, principal component analysis (PCA), and orthogonal projections to latent structures (oPLS) [23,51,91,92], clustering techniques offer unique advantages, especially for large datasets [6,35,93,94]. K-means categorizes participants into distinct non-overlapping groups, while hierarchical clustering captures gradients in smaller datasets or consumption of specific food types [94]. Together, these clustering methods revealed latent, complex patterns in metabolite interactions and provide a complementary perspective to traditional targeted approaches. Our analysis identified taurine and sucrose as key cluster-differentiating metabolites, which likely reflect differences in habitual dietary patterns. Taurine is a sulfur-containing amino acid primarily obtained from animal-based foods such as meat and seafood. Its levels in urine are considered reflective of recent intake and are also influenced by endogenous synthesis and bile acid conjugation pathways. Higher urinary taurine concentrations may indicate increased consumption of animal proteins and have been proposed as biomarkers for Western-type dietary patterns [95,96]. Sucrose, a disaccharide composed of glucose and fructose, is commonly used as a potential marker for dietary sugar intake, particularly when excreted unmetabolized in urine. Its urinary presence reflects incomplete absorption or very high intake and is associated with processed food consumption and sweetened beverages [97,98]. These distinct metabolic signatures support the hypothesis that dietary habits leave measurable imprints on the urinary metabolome and help characterize cluster-specific intake profiles.

Cluster 2, with low levels of taurine and sucrose, likely represents a diet low in sugary foods and poultry. Linear regression showed no significant association between taurine levels and red, fresh, or processed meat intake but revealed a strong positive association with poultry consumption. This suggests that taurine levels in our cohort primarily reflect poultry intake rather than general meat consumption, aligning with nutritional profiles in Cluster 2. Elevated sucrose levels in Cluster 3 indicate a diet high in processed and sugary foods, with poultry as the main source of taurine [97,98,99]. In contrast, Cluster 4 exhibited low sucrose levels but similar taurine levels to Cluster 3, reflecting a low sugar diet with consistent poultry intake. Furthermore, multinomial regression analysis highlighted dietary differences between the clusters, consistent with the metabolite-related differences observed. Members of Cluster 3 had higher intakes of alcoholic beverages and fruit, corresponding with higher sucrose levels, while processed meat intake was higher in Clusters 3 and 2 compared to Cluster 4, consistent with taurine patterns. These findings reinforce taurine and sucrose as key dietary markers related to the identified urinary metabolite clusters in this cohort. The relative consistency of other metabolites across clusters suggests dietary homogeneity within the Augsburg population. Factors such as hydration status and timing of urine collection are unlikely to account for these similarities, as samples were consistently collected in the fasting state, with concentrations normalized to creatinine. 

The present study has several strengths, including a robust sample size of 496 participants with comprehensive dietary assessments. Dietary data collection included both the myfood24 online platform for repeated 24-h food recalls and an FFQ, providing detailed insights into participants’ habitual intake. However, limitations include potential measurement errors in self-reported dietary data, which may weaken associations with urinary metabolomic data. The cross-sectional design further limits causal inference and does not account for temporal dietary or metabolic changes. The generalizability of the findings is further limited by the regional scope of participant recruitment and the relatively narrow age range of the study population. As our analysis was based exclusively on data from the German population, the results may not be directly transferable to populations with different genetic backgrounds, dietary practices, or environmental exposures. Several of the identified metabolites, such as trimethylamine-N-oxide (TMAO), indoxyl sulfate (IS), and hippurate, are end products of gut microbial fermentation and are strongly influenced by microbiota composition, which varies substantially between populations due to differences in genetics, cultural dietary practices, and environmental exposures [42,43,50,55,100]. Consequently, biomarker patterns observed in our study may differ in cohorts with distinct gut microbiota profiles, affecting both the interpretation and potential application of urinary metabolite markers across different populations. Future studies in more diverse populations are needed to validate and expand upon our findings. In addition, the study lacks data on microbiota composition and genetic factors, which could have improved biomarker identification by accounting for individual variability in metabolite levels. Another limitation is the applied handling of unknown data (NaN). To avoid loss of participants, we coded NaNs as values below the detection limit. According to the manufacturer’s comment, NaNs could be very low or very high metabolite concentrations. Most NaNs were identified for ethanol and sucrose values. As we used spot urine samples collected in the morning following a 12-h fasting period, we concluded that very high values of ethanol or sucrose are unrealistic. However, as only a single measurement was available, the metabolite data represent a snapshot of dietary intake from the previous day. In addition, the planned sensitivity analysis could not be performed since the exclusion of all NaNs drastically reduced the sample size for cluster analysis. Furthermore, information on the consumption of taurine-enriched beverages, such as energy drinks, was not collected and should be considered a limitation. Given that taurine was among the investigated metabolites, the lack of data on specific sources like energy drinks may have affected the interpretation of urinary taurine levels in relation to habitual dietary intake.

## 5. Conclusions

Our findings highlight associations between habitual diet and urinary metabolite profiles, confirming known associations for citrus, protein, and fiber intake, while also identifying new potential markers for poultry and milk substitute intake, as well as for dietary fiber intake. Future research should use longitudinal approaches with sufficiently large numbers of participants, incorporate microbiota and genetic data, and validate these biomarkers in diverse populations to more accurately capture habitual diet–metabolite relationships.

## Figures and Tables

**Table 1:** Characteristics of the total study population and stratified by sex.

Characteristics	Total (*n* = 496)		Male (*n* = 211)		Female (*n* = 285)		*p*-Value
	Mean (SD)	Median (IQR)	Mean (SD)	Median (IQR)	Mean (SD)	Median (IQR)	
Age (y)	47.33 (14.64)	49.00 (26.00)	47.81 (15.10)	50.00 (27.00)	46.98 (14.31)	49.00 (23.00)	0.430 ^b^
BMI (kg/m^2^)	26.27 (5.13)	25.56 (6.26)	27.38 (4.43)	26.93 (5.13)	25.45 (5.46)	24.10 (6.78)	<0.001 ^b^
Waist circumference (cm)	87.84 (15.16)	87.00 (24.00)	96.00 (13.56)	96.00 (18.00)	81.81 (13.38)	79.00 (19.00)	<0.001 ^b^
Cholesterol (mg/dL)	194.94 (38.67)	194.00 (54.00)	190.13 (38.98)	189.00 (54.50)	198.54 (38.10)	196.00 (56.00)	0.017 ^a^
LDL-C (mg/dL)	118.81 (33.70)	117.00 (47.00)	120.00 (34.04)	120.00 (44.50)	117.91 (33.47)	116.00 (50.00)	0.497 ^a^
HDL-C (mg/dL)	62.96 (16.81)	61.00 (22.00)	54.68 (13.54)	52.00 (19.00)	69.16 (16.35)	67.50 (21.00)	<0.001 ^b^
Dietary Protein (g)	69.35 (26.91)	65.55 (32.69)	81.48 (29.59)	78.06 (33.53)	60.37 (20.61)	57.53 (25.80)	<0.001 ^b^
Dietary Fat (g)	74.40 (30.84)	69.81 (40.45)	84.31 (33.35)	83.41 (41.02)	65.33 (26.11)	63.22 (34.78)	<0.001 ^b^
Dietary Carbohydrates (g)	188.41 (66.90)	179.67 (79.32)	215.40 (70.28)	207.59 (78.64)	168.43 (56.64)	164.47 (68.83)	<0.001 ^b^
Dietary Fiber (g)	18.77 (8.64)	17.00 (10.26)	19.54 (9.51)	16.95 (11.49)	18.20 (7.90)	17.08 (10.20)	<0.001 ^b^
Dietary Energy (kcal)	1775.66 (593.57)	1728.26 (700.45)	2072.31 (618.20)	1985.50 (781.20)	1556.05 (466.61)	1532.23 (594.69)	<0.001 ^b^
Dietary Energy (kJ)	7435.34 (2485.69)	7239.34 (2969.87)	8678.10 (2588.25)	8313.46 (3266.01)	6515.26 (1954.13)	6413.02 (2490.85)	<0.001 ^b^
	***n*** **(%)**		***n*** **(%)**		***n*** **(%)**		***p*** **-Value**
Smoker:							0.160
Current	76 (15.32%)		35 (16.59%)		41 (14.39%)		
Never	248 (50.00%)		95 (45.02%)		153 (53.68%)		
Previous	172 (34.68%		81 (38.39%)		91 (31.93%)		
PAL:							0.301
Sedentary	139 (28.66%)		31 (15.12%)		56 (20.00%)		
Low Active	154 (31.75%)		61 (29.76%)		93 (33.21%)		
Active	87 (17.94%)		64 (31.22%)		75 (26.79%)		
Very Active	105 (21.65%)		49 (23.90%)		56 (20.00%)		
Risky Alcohol Consumption Pattern:							<0.001
Low	239 (49.08%)		91 (43.96%)		148 (52.86%)		
Moderate	183 (37.58%)		67 (32.37%)		116 (41.43%)		
High	46 (9.45%)		35 (16.91%)		11 (3.93%)		
Severe	19 (3.90%)		14 (6.76%)		5 (1.79%)		

Mean (SD) and Median (IQR) were reported for continuous variables and *n* (column %), while *n* (column %) was used for categorical variables. We applied Student’s *t*-tests ^a^ for normally distributed continuous variables and Mann–Whitney U-tests ^b^ for non-normally distributed continuous variables, and Chi-square tests for categorical variables. Corresponding *p*-values were used to evaluate statistical significance.

**Table 2:** Habitual food consumption in the study population and stratified by sex.

Food Groups	Total (*n* = 496)		Male (*n* = 211)		Female (*n* = 285)		*p*-Value
(g/Day)	Mean (SD)	Median (IQR)	Mean (SD)	Median (IQR)	Mean (SD)	Median (IQR)	
Fresh Meat	31.60 (51.45)	0.00 (46.82)	39.53 (60.77)	0.00 (63.48)	25.73 (42.46)	0.00 (35.71)	0.084
Processed Meat	32.18 (48.20)	14.29 (46.43)	45.87 (57.21)	28.57 (71.08)	22.05 (37.23)	8.57 (30.00)	<0.001
Fish and Fish Products	11.12 (28.43)	0.00 (4.71)	14.22 (36.29)	0.00 (7.14)	8.83 (20.57)	0.00 (4.29)	0.819
Eggs	8.77 (17.50)	0.00 (10.11)	9.14 (18.25)	0.00 (9.99)	8.49 (16.95)	0.00 (10.00)	0.882
Milk and Dairy Products	106.57 (115.16)	71.43 (126.99)	114.57 (135.57)	69.29 (146.42)	100.64 (97.16)	76.68 (120.73)	0.881
Butter	6.55 (8.07)	4.29 (8.72)	7.83 (9.38)	5.14 (11.61)	5.60 (6.81)	4.00 (7.15)	0.057
Other Edible Fats/Oils	5.26 (9.06)	1.82 (6.36)	5.27 (10.41)	1.14 (4.62)	5.25 (7.93)	2.11 (7.41)	0.008
Bread and Bakery Products	107.98 (77.21)	95.00 (100.97)	126.72 (87.42)	115.00 (106.84)	94.10 (65.46)	86.63 (88.81)	<0.001
Staple Food	61.54 (76.81)	34.23 (90.01)	63.62 (82.22)	35.71 (93.91)	60.00 (72.66)	33.67 (88.85)	0.735
Whole Grain Products	35.94 (50.36)	14.29 (53.93)	41.23 (60.65)	11.43 (66.67)	32.02 (40.80)	16.67 (46.43)	0.842
Potatoes	22.47 (46.41)	0.00 (24.40)	23.32 (50.36)	0.00 (20.36)	21.84 (43.33)	0.00 (26.57)	0.535
Vegetables	88.25 (97.34)	58.57 (112.75)	76.27 (104.77)	44.36 (100.78)	97.12 (90.62)	72.75 (110.87)	<0.001
Legumes	6.90 (27.09)	0.00 (0.00)	6.78 (27.57)	0.00 (0.00)	6.99 (26.78)	0.00 (0.00)	0.768
Fruits	100.72 (108.23)	70.72 (139.38)	82.58 (108.95)	43.43 (125.95)	114.15 (105.90)	90.00 (131.14)	<0.001
Nuts	10.11 (18.33)	0.00 (13.72)	9.80 (19.30)	0.00 (12.74)	10.34 (17.61)	0.00 (14.29)	0.194
Sweets	20.88 (27.45)	10.71 (31.11)	22.27 (31.09)	9.29 (30.44)	19.85 (24.41)	12.86 (31.43)	0.850
Non-Alcoholic Beverages	1382.40 (808.89)	1307.14 (983.16)	1472.31 (854.85)	1367.86 (1064.55)	1315.83 (767.86)	1261.35 (926.05)	0.048
Alcoholic Beverages	200.31 (338.81)	45.53 (266.49)	352.36 (446.18)	176.43 (622.50)	87.73 (151.60)	14.29 (107.14)	<0.001
Roasted Coffee	283.12 (235.24)	241.36 (300.00)	274.89 (254.52)	232.14 (348.22)	289.21 (220.13)	250.00 (264.29)	0.156
Soups and Sauces	55.61 (95.30)	4.22 (80.00)	57.00 (96.18)	0.00 (85.71)	54.58 (94.80)	5.71 (71.43)	0.752
Meat and Milk Alternatives	19.81 (60.32)	0.00 (0.00)	16.47 (59.74)	0.00 (0.00)	22.28 (60.74)	0.00 (2.14)	0.006

Data are presented as Mean (SD) and Median (IQR) in grams per day (g/day) for each food group. We applied Mann–Whitney U-tests for non-normally distributed continuous variables to assess differences between groups. Due to the classification approach, where each food item was assigned to only one food group, even if it could belong to multiple categories, the median intake of certain groups (e.g., eggs or meat and milk alternatives) may not be fully representative.

**Table 3:** Urinary metabolite concentrations, expressed in (mmol/mmol creatinine) × 100, overall and stratified by sex.

Abbreviations	Metabolites	Total (*n* = 496)		Male (*n* = 211)		Female (*n* = 285)		*p*-Value
		Mean (SD)	Median (IQR)	Mean (SD)	Median (IQR)	Mean (SD)	Median (IQR)	
(mmol/mmol Creatine) × 100								
ACE rCr	Acetate	1.83 (24.89)	0.49 (0.53)	3.14 (37.94)	0.36 (0.40)	0.85 (0.87)	0.60 (0.67)	<0.001 ^b^
ALA rCr	Alanine	1.88 (0.84)	1.71 (0.91)	1.94 (0.84)	1.82 (1.18)	1.83 (0.83)	1.68 (0.78)	0.153 ^b^
ALN rCr	Allantoin	4.26 (40.28)	0.59 (0.72)	3.88 (43.67)	0.73 (0.75)	4.54 (37.62)	0.52 (0.59)	<0.001 ^b^
AOHIBUT rCr	2-Hydroxyisobutyrate	0.51 (0.15)	0.50 (0.19)	0.50 (0.14)	0.48 (0.18)	0.51 (0.15)	0.51 (0.20)	0.209 ^a^
ARB rCr	Arabinose	0.54 (0.41)	0.45 (0.33)	0.50 (0.46)	0.41 (0.27)	0.58 (0.36)	0.50 (0.36)	<0.001 ^b^
BNHIBUT rCr	3-Aminoisobutyrate	8.36 (74.61)	0.65 (1.16)	8.09 (71.86)	0.53 (1.12)	8.56 (76.75)	0.73 (1.13)	0.042 ^b^
BOHIBUT rCr	3-Hydroxyisobutyrate	0.77 (0.35)	0.70 (0.35)	0.77 (0.30)	0.71 (0.35)	0.77 (0.38)	0.70 (0.37)	0.418 ^b^
BOHIVA rCr	3-Hydroxyisovalerate	0.54 (1.67)	0.41 (0.25)	0.64 (2.52)	0.43 (0.23)	0.46 (0.27)	0.40 (0.25)	0.161 ^b^
CACO rCr	cis-Aconitate	1.97 (0.78)	1.83 (0.85)	1.76 (0.83)	1.65 (0.57)	2.12 (0.70)	2.03 (0.87)	<0.001 ^b^
CIT rCr	Citrate	22.47 (12.32)	20.81 (16.09)	15.39 (8.22)	14.37 (10.58)	27.85 (12.21)	27.32 (16.31)	<0.001 ^b^
CREA rCr	Creatinine	10.19 (6.75)	9.06 (9.89)	11.69 (6.91)	10.79 (9.62)	9.06 (6.41)	7.68 (9.03)	<0.001 ^b^
DMA rCr	Dimethylamine	3.09 (0.80)	2.99 (0.63)	2.92 (0.70)	2.78 (0.56)	3.21 (0.85)	3.11 (0.55)	<0.001 ^b^
DOETA rCr	4-Deoxyerythronic acid	0.77 (0.36)	0.69 (0.40)	0.74 (0.30)	0.68 (0.36)	0.79 (0.40)	0.70 (0.43)	0.643 ^b^
DTA rCr	4-Deoxythreonate	2.30 (0.97)	2.14 (1.10)	2.59 (1.03)	2.40 (1.26)	2.08 (0.86)	1.96 (1.01)	<0.001 ^b^
ETNH rCr	Ethanolamine	4.32 (1.49)	4.21 (1.90)	3.95 (1.27)	3.89 (1.78)	4.60 (1.58)	4.52 (1.96)	<0.001 ^a^
ETOH rCr	Ethanol	10.96 (91.73)	0.19 (0.32)	8.03 (83.70)	0.15 (0.25)	13.12 (97.40)	0.24 (0.35)	0.002 ^b^
FORM rCr	Formate	1.59 (0.84)	1.52 (1.00)	1.48 (0.82)	1.33 (1.01)	1.68 (0.84)	1.62 (1.05)	0.002 ^b^
FURGL rCr	2-Furoylglycine	18.89 (106.47)	0.13 (0.17)	23.50 (124.05)	0.15 (0.17)	15.27 (90.38)	0.11 (0.18)	0.487 ^b^
GLC rCr	Glucose	7.66 (65.92)	2.97 (1.54)	12.60 (100.33)	2.59 (0.95)	3.93 (2.67)	3.51 (1.65)	<0.001 ^b^
GLN rCr	Glutamine	3.69 (29.84)	2.05 (2.02)	2.34 (1.57)	2.00 (1.87)	4.72 (39.56)	2.10 (2.26)	0.980 ^b^
GLY rCr	Glycine	9.52 (6.56)	8.00 (5.94)	7.51 (4.12)	6.49 (4.42)	11.02 (7.57)	9.16 (7.26)	<0.001 ^b^
GLYA rCr	Glycolic acid	4.15 (2.19)	3.80 (2.41)	4.20 (2.11)	3.79 (2.32)	4.11 (2.25)	3.81 (2.50)	0.731 ^b^
HIP rCr	Hippurate	31.18 (26.51)	23.23 (24.43)	25.65 (22.06)	20.05 (21.06)	35.36 (28.77)	27.47 (27.11)	<0.001 ^b^
HPHPA rCr	3-(3-Hydroxyphenyl)-3-Hydroxypropionic acid	2.55 (2.26)	1.71 (2.51)	2.27 (2.20)	1.41 (1.95)	2.75 (2.29)	2.01 (2.75)	0.004 ^b^
HYP rCr	Hypoxanthine	0.94 (0.53)	0.85 (0.54)	0.87 (0.55)	0.78 (0.47)	0.99 (0.51)	0.92 (0.51)	0.001 ^b^
ILE rCr	Isoleucine	2.52 (41.60)	0.09 (0.08)	4.43 (61.08)	0.08 (0.06)	1.09 (15.86)	0.10 (0.11)	<0.001 ^b^
IND rCr	Indoxyl Sulfate	2.73 (1.37)	2.50 (1.67)	2.40 (1.11)	2.19 (1.37)	2.98 (1.49)	2.79 (1.74)	<0.001 ^b^
LAC rCr	Lactate	1.33 (1.65)	0.89 (0.94)	0.81 (1.69)	0.65 (0.45)	1.71 (1.52)	1.25 (1.34)	<0.001 ^b^
LEU rCr	Leucine	0.18 (0.07)	0.17 (0.09)	0.18 (0.07)	0.17 (0.07)	0.18 (0.08)	0.17 (0.09)	0.519 ^b^
MNT rCr	Mannitol	5.28 (41.82)	1.18 (3.08)	7.51 (63.40)	0.99 (2.43)	3.59 (4.99)	1.30 (4.10)	0.231 ^b^
MOHHIP rCr	3-Hydroxyhippurate	2.05 (1.94)	1.39 (1.99)	1.91 (1.94)	1.24 (1.79)	2.15 (1.93)	1.49 (2.24)	0.082 ^b^
OMNA rCr	1-Methylnicotinamide	0.70 (0.37)	0.62 (0.41)	0.60 (0.28)	0.55 (0.34)	0.78 (0.41)	0.69 (0.46)	<0.001 ^b^
PGLU rCr	Pyroglutamate	2.35 (0.70)	2.26 (0.77)	2.27 (0.75)	2.10 (0.71)	2.41 (0.64)	2.35 (0.77)	<0.001 ^b^
POHHIP rCr	4-Hydroxyhippurate	1.40 (1.27)	1.06 (0.82)	1.35 (1.44)	0.96 (0.78)	1.43 (1.12)	1.14 (0.81)	0.004 ^b^
PRGLY rCr	Propylene Glycol	6.30 (61.79)	0.40 (0.46)	3.49 (39.47)	0.40 (0.52)	8.35 (73.89)	0.40 (0.41)	0.888 ^b^
PROBET rCr	Proline Betaine	6.37 (63.18)	0.67 (1.26)	2.02 (11.64)	0.61 (1.17)	9.68 (83.12)	0.71 (1.36)	0.352 ^b^
PSEUR rCr	Pseudouridine	3.04 (0.49)	3.03 (0.57)	2.84 (0.42)	2.82 (0.49)	3.19 (0.47)	3.17 (0.54)	<0.001 ^a^
QUINA rCr	Quinic acid	2.60 (2.00)	2.22 (2.62)	2.17 (1.79)	1.79 (1.98)	2.93 (2.09)	2.62 (2.76)	<0.001 ^b^
SCR rCr	Sucrose	72.68 (197.82)	0.23 (0.49)	88.40 (204.72)	0.24 (1.39)	58.33 (190.74)	0.21 (0.34)	0.294 ^b^
TACO rCr	trans-Aconitate	0.48 (0.29)	0.44 (0.21)	0.48 (0.20)	0.45 (0.20)	0.48 (0.34)	0.43 (0.21)	0.282 ^b^
TAU rCr	Taurine	7.25 (38.37)	3.61 (5.45)	5.49 (5.92)	3.86 (5.01)	8.82 (52.44)	3.16 (5.71)	0.048 ^b^
THRE rCr	Threonine	0.65 (0.40)	0.58 (0.43)	0.68 (0.40)	0.61 (0.42)	0.63 (0.40)	0.55 (0.44)	0.075 ^b^
TMAO rCr	Trimethylamine-N-oxide	4.67 (4.81)	3.56 (2.59)	4.50 (4.19)	3.24 (2.61)	4.81 (5.23)	3.65 (2.60)	0.161 ^b^
TRIG rCr	Trigonelline	3.48 (2.81)	2.85 (3.26)	2.88 (2.75)	2.08 (2.84)	3.94 (2.77)	3.31 (3.59)	<0.001 ^b^
TRP rCr	Tryptophan	3.58 (48.15)	0.57 (0.33)	7.55 (73.70)	0.56 (0.31)	0.63 (0.30)	0.57 (0.35)	0.452 ^b^
TYR rCr	Tyrosine	1.03 (0.51)	0.96 (0.65)	1.09 (0.50)	1.04 (0.68)	0.98 (0.51)	0.88 (0.61)	0.003 ^b^
URA rCr	Uracil	0.59 (0.28)	0.53 (0.31)	0.51 (0.26)	0.46 (0.25)	0.64 (0.28)	0.59 (0.34)	<0.001 ^b^
VAL rCr	Valine	0.23 (0.09)	0.21 (0.11)	0.22 (0.09)	0.20 (0.12)	0.23 (0.09)	0.22 (0.10)	0.020 ^b^
XAN rCr	Xanthosine	0.93 (0.22)	0.89 (0.23)	0.87 (0.21)	0.84 (0.19)	0.98 (0.22)	0.96 (0.23)	<0.001 ^b^
XYL rCr	Xylose	0.74 (0.44)	0.68 (0.36)	0.74 (0.59)	0.64 (0.33)	0.73 (0.30)	0.70 (0.36)	0.094 ^b^

Metabolite concentrations are presented as Mean (SD) and Median (IQR) in units of 100 mmol/L, adjusted relative to creatinine. We applied Student’s *t*-tests ^a^ for normally distributed continuous variables, Mann–Whitney U-tests ^b^ for non-normally distributed continuous variables. Corresponding *p*-values were used to evaluate statistical significance.

**Table 4:** Multivariable-adjusted linear and median regression analyses on the associations between habitual dietary intake and selected urinary metabolites (dependent variable).

Food Groups/Items	Metabolites	Estimate	Lower CI	Upper CI	*p*-Value
CITRUS FRUITS					
Citrus Fruits	PROBET rCr	0.0054	0.0016	0.0093	0.0061 ^c^
DIETARY PROTEIN					
Dietary Protein	URA rCr	0.0789	0.0281	0.1297	0.0024 ^c^
MEAT					
Fresh Meat	TAU rCr	0.0035	0.0007	0.0063	0.0145 ^d^
Red Meat	TAU rCr	0.0021	−0.0024	0.0067	0.3599 ^d^
Red Meat	TMAO rCr	0.0009	−0.0005	0.0024	0.2217 ^c^
Poultry	TAU rCr	0.0052	0.0021	0.0084	0.0010 ^d^
Poultry	IND rCr	0.0021	0.0006	0.0037	0.0069 ^c^
Poultry	OMNA rCr	0.0014	0.0001	0.0027	0.0326 ^c^
Poultry	TMAO rCr	0.0022	0.0005	0.0039	0.0094 ^c^
FISH					
Fish	DMA rCr	−0.0003	−0.0011	0.0005	0.4603 ^c^
Fish	TMAO rCr	−0.0020	−0.0046	0.0007	0.1409 ^c^
Fish	TAU rCr	0.0025	−0.0091	0.0143	0.6648 ^d^
MILK SUBSTITUTES					
Milk Substitutes	URA rCr	0.0009	0.0001	0.0017	0.0163 ^c^
Milk Substitutes	PSEUR rCr	0.0010	0.0003	0.0017	0.0036 ^c^
Milk Substitutes	MOHHIP rCr	0.0019	0.0002	0.0036	0.0221 ^d^
Milk Substitutes	POHHIP rCr	0.0016	0.0001	0.0030	0.0316 ^d^
Milk Substitutes	QUINA rCr	−0.0025	−0.0046	−0.0003	0.0224 ^d^
DIETARY FIBER					
Dietary Fiber	ACE rCr	0.0092	−0.0013	0.0198	0.0866 ^d^
Dietary Fiber	BNHIBUT rCr	0.0170	−0.0003	0.0376	0.1042 ^d^
Dietary Fiber	BOHIBUT rCr	0.0003	−0.0049	0.0056	0.9062 ^d^
Dietary Fiber	HPHPA rCr	0.0233	0.0055	0.0412	0.0105 ^d^
Dietary Fiber	HIP rCr	0.0137	0.0060	0.0215	0.0004 ^c^
Dietary Fiber	IND rCr	−0.0066	−0.0128	−0.0003	0.0390 ^c^
Dietary Fiber	FORM rCr	0.0034	−0.0046	0.0115	0.4056 ^c^
Dietary Fiber	AOHIBUT rCr	−0.0009	−0.0024	0.0006	0.2580 ^c^
Dietary Fiber	BOHIVAL rCr	0.0034	−0.0037	0.0106	0.3521 ^c^

Associations between food groups and specific metabolites (as ratios of metabolite concentration by creatinine concentration) are presented using log-transformed metabolite outcomes, except for URE, PSEUR, and AOHIBUT, which are normally distributed. Results, including estimates, confidence intervals, and *p*-values, account for the log-normal distribution of the data where applicable. Associations were derived from linear ^c^ and median ^d^ regression models.

## Data Availability

The raw data supporting the conclusions of this article will be made available by the authors upon request.

## Supplementary Materials

The following supporting information can be downloaded at: https://www.mdpi.com/article/10.3390/metabo15070441/s1, Table S1: Characteristic of the study population by cluster; Table S2: Urinary metabolite concentrations by cluster (excluding Cluster 1 due to missing data in the non-log-transformed dataset); Table S3. Significant linear associations between food groups and clusters of metabolites; Figure S1. Partial effect plot of non-linear association between fruit intake and cluster membership: Cluster 3 vs. Cluster 4; Figure S2. Partial effect plot of non-linear association between processed meat intake and cluster membership: Cluster 3 vs. Cluster 4; Figure S3. Partial effect plot of non-linear association between poultry intake and cluster membership: Cluster 2 vs. Cluster; Figure S4. Partial effect plot of non-linear association between poultry intake and cluster membership: Cluster 3 vs. Cluster 2.

## Abbreviations

The following abbreviations are used in this manuscript:

ACE	Angiotensin Converting Enzyme
AIC	Akaike Information Criterion
BMI	Body Mass Index
EHIS-PAQ	European Health Interview Survey-Physical Activity Questionnaire
HPHPA	3-(3-hydroxyphenyl)-3-hydroxypropionic acid
2-HPPA	3-(2-hydroxyphenyl)-propionic acid
IQR	Interquartile Range
IS	Indoxyl Sulphate
MEIA	Metabolism, Nutrition, and Immune System in Augsburg Study
NCD	Non-Communicable Disease
N1-MN	1-Methylnicotinamide
oPLS	orthogonal Projection to Latent Structures
PAL	Physical Activity Level
PCA	Principal Component Analysis
RNA	Ribonucleic Acid
SCFA	Short-Chain Fatty Acid
SD	Standard Deviation
TMAO	Trimethylamine-N-oxide
VIF	Variance Inflation Factor
VIP	Variable in Projection
