# Supplementary material for: Association Between Habitual Dietary Intake and Urinary Metabolites in Adults—Results of a Population-Based Study
Source: Metabolites. 2025 Jul 1;15(7):441. doi: 10.3390/metabo15070441 (PMC12297984; doi:10.3390/metabo15070441)
Supplement: Supplementary file 1 [file metabolites-15-00441-s001.zip › metabolites-3644411-supplementary.pdf]

Supplementary Material

Supplementary Table S1. Characteristic of the study population by cluster.

Characteristics	Total (n=496)		Cluster 1 (n=10)		Cluster 2 (n=84)		Cluster 3 (n=56)		Cluster 4 (n=346)		*p*-Value
	Mean (SD)	Median (IQR)	Mean (SD)	Median (IQR)	Mean (SD)	Median (IQR)	Mean (SD)	Median (IQR)	Mean (SD)	Median (IQR)	
**Age (y)**	47.33 (14.64)	49.00 (14.64)	46.60 (15.41)	47.00 (15.41)	48.51 (13.75)	51.50 (13.75)	47.34 (15.98)	47.00 (15.98)	47.07 (14.65)	49.00 (14.65)	0.915[b]
**BMI (kg/m²)**	26.27 (5.13)	25.56 (5.13)	27.60 (10.64)	24.57 (10.64)	25.22 (4.63)	24.59 (4.63)	26.43 (3.88)	26.27 (3.88)	26.46 (5.19)	25.65 (5.19)	0.123[b]
**Waist circum-ference (cm)**	87.84 (15.16)	87.00 (15.16)	89.90 (21.52)	92.50 (21.52)	83.77 (13.19)	81.00 (13.19)	89.79 (12.99)	88.50 (12.99)	88.46 (15.62)	88.00 (15.62)	0.058[b]
**Cholesterol (mg/dL)**	194.94 (38.67)	194.00 (38.67)	186.22 (33.84)	178.00 (33.84)	201.00 (41.02)	198.50 (41.02)	189.82 (37.79)	194.50 (37.79)	194.52 (38.29)	193.00 (38.29)	0.316[a]
**LDL-C (mg/dL)**	118.81 (33.70)	117.00 (33.70)	118.00 (27.11)	106.00 (27.11)	120.99 (35.76)	115.50 (35.76)	116.62 (33.23)	116.00 (33.23)	118.65 (33.52)	118.00 (33.52)	0.897[a]
**HDL-C (mg/dL)**	62.96 (16.81)	61.00 (16.81)	53.11 (13.71)	53.00 (13.71)	67.23 (17.28)	64.50 (17.28)	60.43 (15.05)	59.00 (15.05)	62.59 (16.86)	61.00 (16.86)	0.027[b]
**Dietary Protein (g)**	69.35 (26.91)	65.55 (26.91)	75.83 (33.06)	61.65 (33.06)	63.89 (22.45)	64.81 (22.45)	78.00 (26.48)	74.36 (26.48)	69.09 (27.51)	63.49 (27.51)	0.019[b]
**Dietary Fat (g)**	73.40 (30.84)	69.81 (30.84)	87.38 (41.61)	67.21 (41.61)	67.00 (27.54)	61.19 (27.54)	80.62 (29.57)	78.82 (29.57)	73.38 (31.21)	69.19 (31.21)	0.032[b]
**Dietary Carbohydrates (g)**	188.41 (66.90)	179.67 (66.90)	212.42 (71.89)	199.05 (71.89)	185.15 (67.09)	180.92 (67.09)	197.51 (61.17)	199.18 (61.17)	187.04 (67.63)	175.15 (67.63)	0.236[b]
**Dietary Fiber (g)**	18.77 (8.64)	17.00 (8.64)	19.36 (10.06)	15.90 (10.06)	18.66 (8.85)	16.77 (8.85)	19.73 (8.77)	18.08 (8.77)	18.62 (8.55)	16.89 (8.55)	0.716[b]
**Dietary Energie (kcal)**	1775.66 (593.57)	1728.26 (593.57)	1978.10 (786.92)	1618.63 (786.92)	1674.95 (531.66)	1631.03 (531.66)	1948.23 (592.28)	1957.17 (592.28)	1766.34 (597.38)	1719.74 (597.38)	0.033[b]
**Dietary Energie (kJ)**	7435.34 (2485.69)	7239.34 (2485.69)	8282.75 (3297.47)	6774.03 (3297.47)	7014.34 (2228.53)	6831.92 (2228.53)	8156.43 (2479.51)	8196.76 (2479.51)	7396.35 (2501.31)	7196.60 (2501.31)	0.033[b]

	n (%)	n (%)	n (%)	n (%)	n (%)	
**Sex**						< 0.001
Male	211 (42.54%)	2 (20.00%)	18 (21.43%)	38 (67.86%)	153 (44.22%)	
Female	285 (57.46%)	8 (80.00%)	66 (78.57%)	18 (32.14%)	193 (55.78%)	
**Smoker**						0.196
Current	76 (15.32%)	0 (0.00%)	14 (16.67%)	9 (16.07%)	53 (15.32%)	
Never	248 (50.00%)	8 (80.00%)	36 (42.86%)	23 (41.07%)	181 (52.31%)	
Previous	172 (34.68%)	2 (20.00%)	34 (40.48%)	24 (42.86%)	112 (32.37%)	
**PAL**						0.308
Sedentary	87 (17.94%)	3 (33.33%)	12 (14.29%)	10 (18.52%)	62 (18.34%)	
Low Active	154 (31.75%)	5 (55.56%)	22 (26.19%)	19 (35.19%)	108 (31.95%)	
Active	139 (28.66%)	0 (0.00%)	27 (32.14%)	12 (22.22%)	100 (29.59%)	
Very Active	105 (21.65%)	1 (11.11%)	23 (27.38%)	13 (24.07%)	68 (20.12%)	
**Risky Alcohol Consumption Pattern**						0.581
Low Risk	239 (49.08%)	6 (66.67%)	42 (50.60%)	24 (44.44%)	167 (48.97%)	
Moderate Risk	183 (37.58%)	3 (33.33%)	33 (39.76%)	19 (35.19%)	128 (37.54%)	
High Risk	46 (9.45%)	0 (0.00%)	6 (7.23%)	6 (11.11%)	34 (9.97%)	
Severe Risk	19 (3.90%)	0 (0.00%)	2 (2.41%)	5 (9.26%)	12 (3.52%)	

Mean (Standard Deviation, SD) and Median (Interquartile Range, IQR) were reported for continuous variables, while n (column %) was used for categorical variables. P-values were calculated using the ANOVA[a] for normally distributed variables and the Kruskal-Wallis test[b] for non-normally distributed variables. P-values for categorical variables were calculated using the Chi-Square test.

Supplementary Table S2. Urinary metabolite concentrations by cluster (excluding cluster 1 due to missing data in the non-log-transformed dataset).

Abbreviations	Metabolites	Total (n=496)		Cluster 2 (n=84)		Cluster 3 (n=56)		Cluster 4 (n=346)		p-Value
		Mean (SD)	Median (IQR)	Mean (SD)	Median (IQR)	Mean (SD)	Median (IQR)	Mean (SD)	Median (IQR)	
		(mmol/mmol creatine) x 100								
**ACE rCr**	**Acetate**	1.83 (24.89)	0.49 (0.53)	0.72 (0.54)	0.59 (0.61)	0.48 (0.35)	0.39 (0.40)	2.32 (29.51)	0.49 (0.54)	0.025[d]
**ALA rCr**	**Alanine**	1.88 (0.84)	1.71 (0.91)	1.70 (0.71)	1.53 (0.85)	1.68 (0.63)	1.51 (0.83)	1.95 (0.88)	1.81 (0.94)	0.005[d]
**ALN rCr**	**Allantoin**	4.26 (40.28)	0.59 (0.72)	4.33 (32.95)	0.62 (0.73)	0.70 (0.50)	0.58 (0.57)	4.81 (44.94)	0.58 (0.74)	0.878[d]
**AOHIBUT rCr**	**2-Hydroxyisobutyrate**	0.51 (0.15)	0.50 (0.19)	0.51 (0.16)	0.51 (0.16)	0.51 (0.14)	0.49 (0.21)	0.50 (0.15)	0.49 (0.19)	0.890[c]
**ARB**	**Arabinose**	0.54 (0.41)	0.45 (0.33)	0.61 (0.37)	0.54 (0.34)	0.44 (0.23)	0.40 (0.26)	0.54 (0.43)	0.45 (0.33)	0.012[d]
**BNHIBUT rCr**	**3-Aminoisobutyrate**	8.36 (74.61)	0.65 (1.16)	1.45 (2.35)	0.87 (1.04)	2.05 (3.32)	0.75 (1.85)	10.72 (86.76)	0.58 (1.09)	0.096[d]
**BOHIBUT rCr**	**3-Hydroxyisobutyrate**	0.77 (0.35)	0.70 (0.35)	0.74 (0.38)	0.66 (0.33)	0.81 (0.34)	0.69 (0.36)	0.77 (0.34)	0.72 (0.35)	0.303[d]
**BOHIVA rCr**	**3-Hydroxyisovalerate**	0.54 (1.67)	0.41 (0.25)	0.46 (0.35)	0.36 (0.26)	0.52 (0.25)	0.44 (0.23)	0.56 (1.96)	0.41 (0.25)	0.021[d]
**CACO rCr**	**cis-Aconitate**	1.97 (0.78)	1.83 (0.85)	2.21 (0.74)	2.06 (0.79)	1.82 (0.56)	1.68 (0.83)	1.94 (0.81)	1.78 (0.80)	0.001[d]
**CIT rCr**	**Citrate**	22.47 (12.32)	20.81 (16.09)	23.48 (12.15)	22.94 (14.88)	19.28 (11.86)	17.56 (16.60)	22.76 (12.39)	21.57 (16.16)	0.081[d]
**CREA rCr**	**Creatinine**	10.19 (6.75)	9.06 (9.89)	9.43 (6.43)	8.52 (10.55)	12.83 (7.08)	12.35 (9.47)	9.95 (6.70)	8.62 (8.80)	0.006[d]
**DMA rCr**	**Dimethylamine**	3.09 (0.80)	2.99 (0.63)	3.17 (0.54)	3.12 (0.60)	2.87 (0.48)	2.78 (0.52)	3.10 (0.89)	2.98 (0.59)	< 0.001[d]
**DOETA rCr**	**4-Deoxyerythronic acid**	0.77 (0.36)	0.69 (0.40)	0.73 (0.34)	0.66 (0.32)	0.74 (0.29)	0.68 (0.40)	0.78 (0.38)	0.71 (0.41)	0.566[d]

**DTA rCr**	**4-Deoxythreonate**	2.30 (0.97)	2.14 (1.10)	2.21 (1.04)	2.11 (1.25)	2.59 (1.12)	2.55 (1.45)	2.28 (0.92)	2.11 (1.00)	0.046[d]
**ETNH rCr**	**Ethanolamine**	4.32 (1.49)	4.21 (1.90)	4.47 (1.54)	4.46 (2.34)	4.10 (1.35)	4.12 (2.00)	4.33 (1.50)	4.19 (1.76)	0.401[c]
**ETOH rCr**	**Ethanol**	10.96 (91.73)	0.19 (0.32)	28.12 (155.83)	0.27 (0.55)	1.09 (2.58)	0.16 (0.35)	7.51 (71.37)	0.16 (0.28)	0.112[d]
**FORM rCr**	**Formate**	1.59 (0.84)	1.52 (1.00)	1.58 (0.83)	1.55 (0.85)	1.57 (0.80)	1.39 (1.08)	1.60 (0.85)	1.52 (1.03)	0.866[d]
**FURGL rCr**	**2-Furoylglycine**	18.89 (106.47)	0.13 (0.17)	13.60 (64.51)	0.13 (0.17)	13.55 (96.91)	0.07 (0.12)	21.04 (115.67)	0.14 (0.19)	0.004[d]
**GLC rCr**	**Glucose**	7.66 (65.92)	2.97 (1.54)	3.18 (1.18)	2.96 (1.48)	3.17 (1.50)	2.89 (1.15)	9.47 (78.06)	3.02 (1.58)	0.523[d]
**GLN rCr**	**Glutamine**	3.69 (29.84)	2.05 (2.02)	2.27 (1.41)	1.98 (1.96)	2.05 (1.58)	1.83 (1.88)	4.31 (35.36)	2.16 (2.05)	0.201[d]
**GLY rCr**	**Glycine**	9.52 (6.56)	8.00 (5.94)	9.02 (5.44)	7.76 (6.23)	7.66 (3.73)	7.07 (4.06)	9.95 (7.10)	8.26 (6.33)	0.051[d]
**GLYA rCr**	**Glycolic acid**	4.15 (2.19)	3.80 (2.41)	3.69 (1.99)	3.70 (2.36)	3.91 (1.99)	3.73 (1.89)	4.30 (2.25)	3.94 (2.54)	0.052[d]
**HIP rCr**	**Hippurate**	31.18 (26.51)	23.23 (24.43)	33.23 (22.64)	28.17 (31.47)	29.37 (26.99)	22.26 (23.84)	30.98 (27.32)	22.94 (22.40)	0.289[d]
**HPHPA rCr**	**3-(3-Hydroxyphenyl)-3-Hydroxypropionic acid**	2.55 (2.26)	1.71 (2.51)	3.18 (2.72)	2.10 (3.00)	2.17 (1.67)	1.50 (2.03)	2.44 (2.19)	1.69 (2.35)	0.103[d]
**HYP rCr**	**Hypoxanthine**	0.94 (0.53)	0.85 (0.54)	0.95 (0.58)	0.81 (0.61)	0.98 (0.66)	0.85 (0.43)	0.93 (0.49)	0.86 (0.53)	0.922[d]
**ILE rCr**	**Isoleucine**	2.52 (41.60)	0.09 (0.08)	3.40 (29.15)	0.09 (0.11)	0.08 (0.05)	0.08 (0.05)	2.69 (47.10)	0.09 (0.08)	0.030[d]
**IND rCr**	**Indoxyl Sulfate**	2.73 (1.37)	2.50 (1.67)	2.76 (1.44)	2.59 (1.51)	2.44 (1.17)	2.21 (1.63)	2.77 (1.38)	2.51 (1.67)	0.255[d]
**LAC rCr**	**Lactate**	1.33 (1.65)	0.89 (0.94)	1.37 (1.09)	1.02 (1.02)	1.35 (3.22)	0.82 (0.69)	1.31 (1.38)	0.88 (0.93)	0.065[d]
**LEU rCr**	**Leucine**	0.18 (0.07)	0.17 (0.09)	0.17 (0.08)	0.17 (0.09)	0.18 (0.06)	0.17 (0.07)	0.19 (0.07)	0.18 (0.09)	0.200[d]

**MNT rCr**	**Mannitol**	5.28	1.18	15.51	1.16	2.30	0.97	3.41	1.28	0.738[d]
		(41.82)	(3.08)	(101.91)	(5.68)	(2.94)	(2.74)	(5.53)	(2.82)	
**MOHHIP rCr**	**3-Hydroxyhippurate**	2.05	1.39	2.74	1.72	1.72	1.22	1.93	1.36	0.015[d]
		(1.94)	(1.99)	(2.61)	(2.33)	(1.44)	(1.51)	(1.78)	(1.95)	
**OMNA rCr**	**1-Methylnicotinamide**	0.70	0.62	0.77	0.69	0.74	0.69	0.67	0.61	0.067[d]
		(0.37)	(0.41)	(0.42)	(0.54)	(0.34)	(0.47)	(0.36)	(0.37)	
**PGLU rCr**	**Pyroglutamate**	2.35	2.26	2.52	2.34	2.09	2.07	2.35	2.27	0.004[d]
		(0.70)	(0.77)	(0.88)	(0.84)	(0.39)	(0.42)	(0.67)	(0.80)	
**POHHIP rCr**	**4-Hydroxyhippurate**	1.40	1.06	1.41	1.06	1.39	1.04	1.40	1.06	0.896[d]
		(1.27)	(0.82)	(1.20)	(0.91)	(1.55)	(0.54)	(1.24)	(0.85)	
**PRGLY rCr**	**Propylene Glycol**	6.30	0.40	0.78	0.43	0.47	0.33	8.54	0.39	0.357[d]
		(61.79)	(0.46)	(1.15)	(0.38)	(0.41)	(0.42)	(72.89)	(0.46)	
**PROBET rCr**	**Proline Betaine**	6.37	0.67	6.69	0.94	3.94	0.51	6.70	0.68	0.438[d]
		(63.18)	(1.26)	(50.27)	(1.29)	(22.09)	(1.17)	(70.25)	(1.21)	
**PSEUR rCr**	**Pseudouridine**	3.04	3.03	3.13	3.15	2.91	2.83	3.04	3.02	0.034[c]
		(0.49)	(0.57)	(0.46)	(0.49)	(0.52)	(0.68)	(0.48)	(0.56)	
**QUINA rCr**	**Quinic acid**	2.60	2.22	3.04	2.73	2.56	2.14	2.50	2.12	0.103[d]
		(2.00)	(2.62)	(2.23)	(3.35)	(1.62)	(2.89)	(1.99)	(2.47)	
**SCR rCr**	**Sucrose**	72.68	0.23	0.44	0.21	452.69	389.53	0.25	0.17	< 0.001[d]
		(197.82)	(0.49)	(0.84)	(0.29)	(270.70)	(417.44)	(0.29)	(0.23)	
**TACO rCr**	**trans-Aconitate**	0.48	0.44	0.54	0.45	0.49	0.45	0.46	0.44	0.347[d]
		(0.29)	(0.21)	(0.55)	(0.27)	(0.20)	(0.21)	(0.19)	(0.20)	
**TAU rCr**	**Taurine**	7.25	3.61	0.12	0.15	5.83	4.24	7.66	3.58	< 0.001[d]
		(38.37)	(5.45)	(0.09)	(0.14)	(4.74)	(6.40)	(41.51)	(5.40)	
**THRE rCr**	**Threonine**	0.65	0.58	0.59	0.50	0.62	0.57	0.67	0.59	0.144[d]
		(0.40)	(0.43)	(0.38)	(0.44)	(0.39)	(0.42)	(0.41)	(0.43)	
**TMAO rCr**	**Trimethylamine-N-oxide**	4.67	3.56	4.16	3.59	3.57	3.25	4.98	3.59	0.218[d]
		(4.81)	(2.59)	(2.82)	(2.40)	(1.68)	(1.75)	(5.47)	(2.70)	
**TRIG rCr**	**Trigonelline**	3.48	2.85	4.38	3.68	2.95	2.42	3.35	2.59	0.001[d]
		(2.81)	(3.26)	(2.84)	(3.75)	(2.26)	(2.66)	(2.84)	(3.16)	
**TRP rCr**	**Tryptophan**	3.58	0.57	11.79	0.58	0.56	0.51	2.04	0.57	0.400[d]
		(48.15)	(0.33)	(102.57)	(0.34)	(0.21)	(0.24)	(25.98)	(0.34)	

**TYR rCr**	**Tyrosine**	1.03 (0.51)	0.96 (0.65)	0.89 (0.46)	0.81 (0.43)	0.99 (0.52)	0.90 (0.71)	1.07 (0.52)	1.02 (0.68)	0.006[d]
**URA rCr**	**Uracil**	0.59 (0.28)	0.53 (0.31)	0.56 (0.24)	0.50 (0.25)	0.61 (0.30)	0.53 (0.34)	0.59 (0.28)	0.54 (0.32)	0.677[d]
**VAL rCr**	**Valine**	0.23 (0.09)	0.21 (0.11)	0.20 (0.07)	0.19 (0.09)	0.23 (0.09)	0.22 (0.14)	0.23 (0.09)	0.23 (0.11)	0.013[d]
**XAN rCr**	**Xanthosine**	0.93 (0.22)	0.89 (0.23)	0.93 (0.19)	0.91 (0.18)	0.89 (0.23)	0.85 (0.22)	0.94 (0.22)	0.90 (0.23)	0.074[d]
**XYL rCr**	**Xylose**	0.74 (0.44)	0.68 (0.36)	0.77 (0.57)	0.68 (0.32)	0.67 (0.31)	0.60 (0.39)	0.74 (0.43)	0.70 (0.36)	0.221[d]

Metabolite concentrations are presented as Mean (SD) and Median (IQR) in units of 100 mmol/l, adjusted relative to creatinine. P-values were calculated using ANOVA[c] for normally distributed variables or a Kruskal-Wallis test[d] for non-normally distributed variables.

Supplementary Table S3. Significant linear associations between food groups and clusters of metabolites

Food Group	Cluster	OR	Lower CI	Upper CI	*p*-Value
**Cluster 3 vs Cluster 4**					
Alcoholic Beverages	Cluster 3 vs Cluster 4	1,0007	1,0000	1,0013	0,0441
**Cluster 3 vs Cluster 2**					
Alcoholic Beverages	Cluster 3 vs Cluster 2	1,0011	1,0001	1,0021	0,0229
Beer	Cluster 3 vs Cluster 2	1,0014	1,0002	1,0025	0,0195

Odds ratios (ORs), 95% CIs, and p-values for associations between food group intake and cluster membership, estimated using multinomial logistic regression. Only two clusters each were taken from the two multinomial analyses (cluster 2 or cluster 4 as reference category).

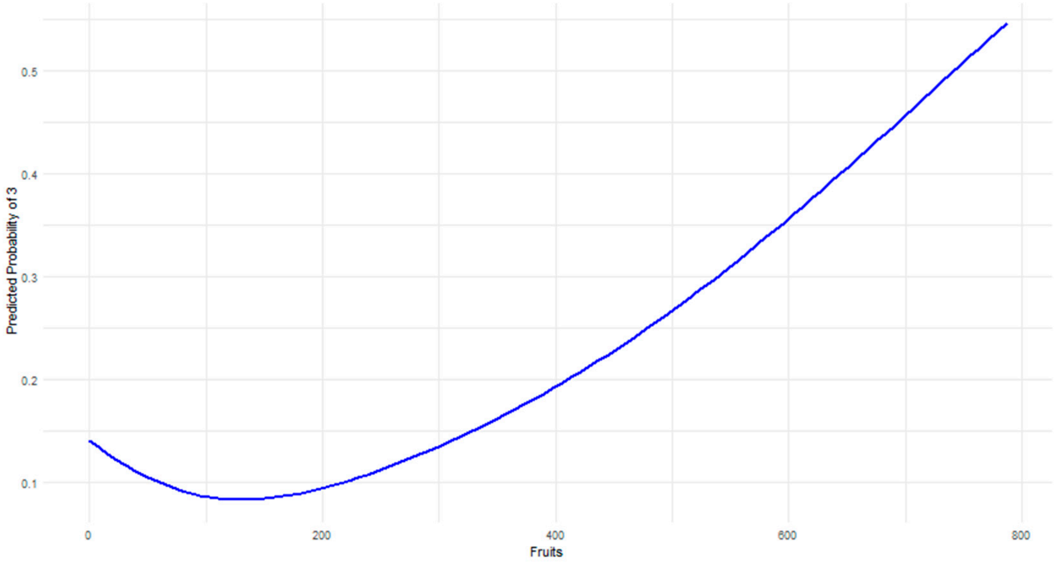

Supplementary Figure S1. Partial effect plot of non-linear association between fruit intake and cluster membership: Cluster 3 vs Cluster 4.

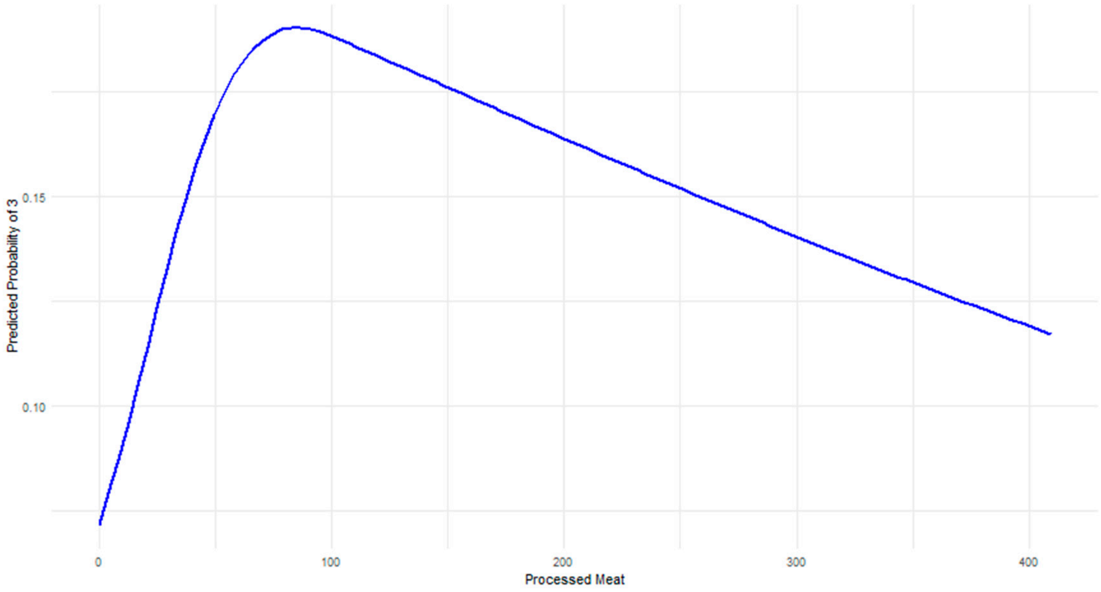

S

Supplementary Figure S2. Partial effect plot of non-linear association between processed meat intake and cluster membership: Cluster 3 vs Cluster 4.

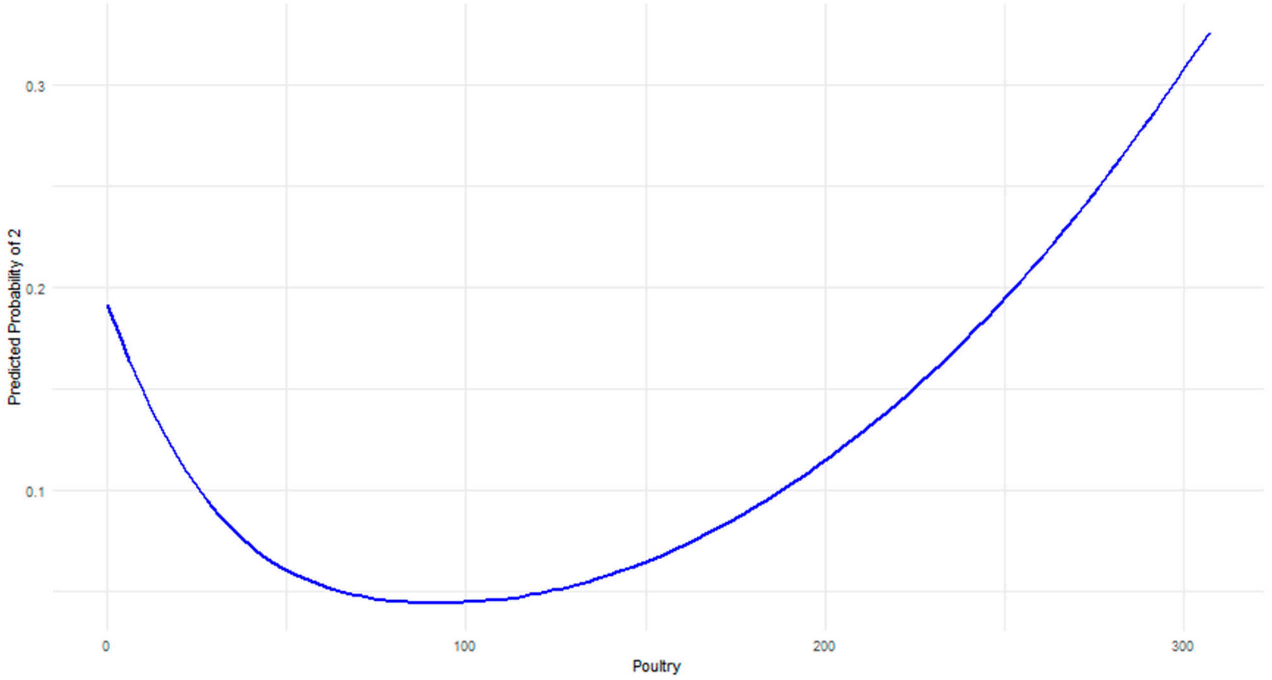

Supplementary Figure S3. Partial effect plot of non-linear association between poultry intake and cluster membership: Cluster 2 vs Cluster 4.

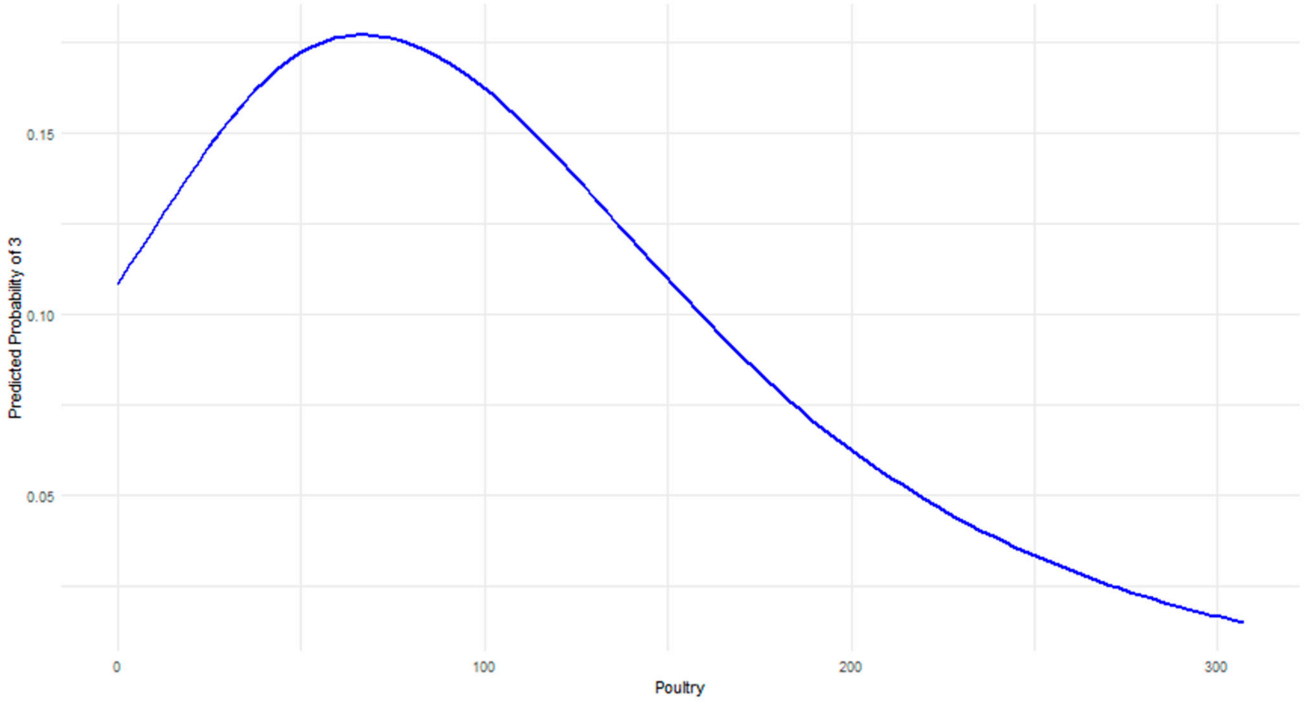

Supplementary Figure S4. Partial effect plot of non-linear association between poultry intake and cluster membership: Cluster 3 vs Cluster 2.
